# Preventive Effect of Collagen Peptides from *Acaudina molpadioides* on Acute Kidney Injury through Attenuation of Oxidative Stress and Inflammation

**DOI:** 10.1155/2022/8186838

**Published:** 2022-05-10

**Authors:** Wei Zhao, Jie Li, Yan Li, Yan Chen, Huoxi Jin

**Affiliations:** Zhejiang Provincial Engineering Technology Research Center of Marine Biomedical Products, School of Food and Pharmacy, Zhejiang Ocean University, Zhoushan 316022, China

## Abstract

The protective effect of collagen peptide from *Acaudina molpadioides* (Amp) on acute kidney injury (AKI) in mice and its mechanism were explored. The results showed that Amp-fed could effectively improve the renal mass index and histopathological morphology. The levels of serum creatinine and urea nitrogen decreased significantly, while the antioxidant enzyme catalase (CAT) and superoxide dismutase (SOD) increased significantly in Amp-fed groups. Western blot results disclosed that Amp significantly upregulates the levels of heme oxygenase-1 (HO-1), Nrf2, p-PI3K, and p-AKT in the kidney. In addition, Amp could significantly downregulate the levels of nuclear factor NF-kappa-B (NF-*κ*B), tumor necrosis factor *α* (TNF-*α*), inflammatory cytokines interleukin 6 (IL-6) and interleukin 1*β* (IL-1*β*). These findings provide evidence that Amp plays a protective role in AKI via attenuation of oxidative stress and inflammation mediated by PI3K/AKT/Nrf2 and PI3K/AKT/NF-*κ*B pathways. This study laid a foundation for the application of Amp in the prevention of AKI.

## 1. Introduction

Acute kidney injury (AKI) is an acute and severe inflammatory process existing in the kidney, which often progresses to chronic kidney disease (CKD) without appropriate treatment. Despite great progress in research into the causes and mechanisms of AKI, it remains a major unmet medical need and will continue to be a global public health problem. Accumulating studies have shown that inflammation and oxidative stress are the key pathways of AKI, affecting its development and severity [1, 2]. To prevent damage caused by oxidative stress, mammalian cells have evolved sophisticated antioxidant defense systems to maintain redox homeostatic and cellular integrity. The core of this self-protective antioxidant defense is the nuclear factor erythrocyte-associated factor 2 (Nrf2), which is anchored in the cytoplasm by Kelch-like epichlorohydrin-related protein-1 (Keap1) in the normal physiological conditions [3]. Free radicals and some antioxidants can stimulate the detachment of Nrf2 from Keap1, resulting in Nrf2 translocation to the nucleus. In the nucleus, the activated Nrf2 binds to the antioxidant response element (ARE), thereby stimulating the expression of antioxidant enzymes such as superoxide dismutase (SOD), heme oxygenase-1 (HO-1), catalase (CAT), and glutathione peroxidase (GSH-Px) [4]. Nrf2 mediates the major cellular defense against cytotoxic effects of oxidative stress and is involved in many physiological and biochemical processes such as antioxidant, detoxification, cell survival, and anti-inflammatory [3–5]. Therefore, the Nrf2-ARE pathway is considered an attractive therapeutic strategy to prevent AKI progression [6].

With the increase of oxidative stress, the inflammatory response occurs through the upregulation of proinflammatory cytokine genes [7, 8]. Nuclear factor-*κ*B (NF-*κ*B) is an important nuclear transcription factor in cells. It participates in the body's inflammatory response and is related to the inflammatory changes in many human diseases such as in liver, kidney, heart, and brain diseases. Therefore, the use of drugs to inhibit the NF-*κ*B signaling pathway may become a means of treatment. It has been proven that the phosphoinositide 3-kinase- (PI3K-) serine/threonine kinase (AKT) pathway is closely related to oxidative stress, inflammation, and cell apoptosis. Previous studies have reported that many compounds could activate the NF-*κ*B and Nrf2-ARE pathway through the PI3K/AKT pathway to protect cells or tissues from damage caused by oxidative stress [8, 9].

Sea cucumber is a precious aquatic product with high protein, low fat, low sugar, and low cholesterol [10]. It was recorded that sea cucumber has a nourishing effect on the liver and kidney [11]. Sea cucumbers are rich in collagen, polysaccharide, saponins, vitamin A, thiamine, and other active substances [12, 13]. The enzymatic hydrolysis products of collagen (collagen peptides) have a wide range of biological activities, such as anticancer, anticoagulant, antioxidant, and antitumor activities [14, 15]. In particular, the marine collagen peptides exhibited better antioxidant effects than mammalian collagen peptides [16]. There are about 140 kinds of sea cucumbers in China, and *Acaudina molpadioides* is one of the 20 edible sea cucumbers [17]. *Acaudina molpadioides* are widely distributed in the East China Sea, and the collagen content in its body wall accounts for more than 70% of the dry weight. However, the collagen of *Acaudina molpadioides* has not been fully utilized and developed, resulting in a great waste of their resources.

It has been proven in our previous study that collagen peptides of *Acaudina molpadioides* (Amp) exhibited good antioxidant activity, thereby effectively protecting RAW264.7 cells from H_2_O_2_-induced damage [18]. However, their ability to protect against AKI and the underlying molecular mechanism has not yet been studied well. In this study, we investigated the role and molecular mechanism of Amp in the prevention of AKI, providing data support for the application of collagen peptides in functional food or pharmacy.

## 2. Materials and Methods

### 2.1. Chemicals and Reagents

Superoxide dismutase (SOD), catalase (CAT), urea nitrogen (BUN), and creatinine (CRE) kits were all purchased from Nanjing Jiancheng Institute of Biological Engineering. The ELISA kits of mouse tumor necrosis factor *α* (TNF-*α*), interleukin-6 (IL-6), interleukin-1*β* (IL-1*β*), nuclear factor Kappa B subunit p105 (NF-*κ*B p105), and all antibodies were purchased from Elabscience Biotechnology Co., Ltd. (Wuhan, China). All other chemicals were from China National Pharmaceutical Group Corporation (Shanghai, China).

### 2.2. Preparation of Collagen Peptides

The collagen peptides of *Acaudina molpadioides* (Amp) were prepared according to the method previously reported in our studies [18].

### 2.3. Experimental Animals

Healthy male ICR mice (18~20 g and 6~8 weeks) were taken from Hangzhou Ziyuan Experimental Animal Science and Technology Co., Ltd. All mice were fed with sterile water and commercial pellet feed. The experiment began after adaptive feeding for one week. The mice were raised and maintained at constant room (temperature: 23 ± 1°C, air humidity: 55% ± 5%) under a normal light/dark schedule (12 h/12 h).

Mice were fed at a fixed time every day (0.2 mL/mouse) and randomly divided into six groups (*n* = 8/group). The control group, the model group, the positive group, and the Amp groups (50, 100, and 200 mg/kg) were given physiological saline, physiological saline, vitamin E (100 mg/kg), and Amp for 21 consecutive days, respectively. After the last administration, the model group, the positive group, and the Amp groups were intraperitoneally injected with 10% CCl_4_ (1 mL/mouse) for 16 h to establish an acute model of kidney injury in mice. All the mice were fed without water for 24 h before being sacrificed. Blood was collected from the eyeball and then centrifuged with 2000 rpm for 10 min to obtain serum. The kidney was removed and stored at -80°C immediately.

### 2.4. Measurement of Renal Mass Index

The kidneys of each group of mice were weighed, and the renal mass index was measured by the following formula:

(1) $$\begin{array}{c} \text{Renal mass index}=\frac{\text{kidney wet weight}}{\text{mouse body weight}}. \end{array}$$

### 2.5. Preparation of Renal Histopathological Examination

The kidneys were fixed overnight with 4% paraformaldehyde, then dehydrated with ethanol, embedded in paraffin, and cut into 3 *μ*m thick sections. Sections were stained with hematoxylin-eosin (H&E) to evaluate the histopathological changes of the kidney. H&E sections were examined for signs of tubular injuries, such as loss of brush boundaries, tubular dilatation, interstitial edema, cell necrosis, and vacuolation.

### 2.6. Renal Function Tests

The tissue homogenate creatinine (CRE) and urea nitrogen (BUN) levels were determined using kits from Nanjing Jiancheng Institute of Biological Engineering.

### 2.7. Detection of Antioxidant Enzymes Activities in Serum

The levels of SOD and CAT in serum were determined according to the instructions of kits (Nanjing Jiancheng Institute of Biological Engineering, China). The SOD and CAT levels were measured at 550 nm with the hydroxylamine method and 412 nm with the colorimetric method by the 1510 spectrophotometer (Thermo Fisher Scientific Oy, Finland), respectively.

### 2.8. Expression of IL-1*β*, IL-6, TNF-*α*, and NF- kB

The levels of IL-1*β*, IL-6, TNF-*α*, and NF-*κ*B were determined by ELISA methods according to the instruction of kits provided by the manufacturer.

### 2.9. Immunohistochemical Analysis

Fresh kidney tissue was fixed with 4% paraformaldehyde and embedded in paraffin. Then, a 3 *μ*m thick section was cut and placed on the slide in the dryer at 37°C overnight. The paraffin-embedded tissue sections were dewaxed in xylene and rehydrated in an ethanol gradient. The slices were then immersed in 0.01 M sodium citrate buffer (pH 6.0) and boiled in the microwave oven. After cooling at room temperature and washing with phosphate-buffered saline (PBS) three times, the sections were incubated with 3% H_2_O_2_ for 10 min and preincubated with goat serum for 1 h at room temperature. The primary antibody against Nrf2 (1 : 200) or HO-1 (1 : 500) was incubated overnight at 4°C and washed in TBST 3 times for 5 min each time. The test was performed using IHC or DAB substrate kit. The water was dehydrated twice for 10 seconds in 95% ethanol, anhydrous ethanol, and xylene. Finally, the sections were sealed and examined under a microscope.

### 2.10. Western Blot Analysis

Renal tissue homogenate was prepared with RIPA buffer and PMSF protein inhibitor. Protein concentration in the kidney was analyzed using the BCA protein assay kit (Beyotime, Shanghai). Protein samples were separated by SDS-PAGE (10%) with an equal amount of protein (30 *μ*g). The protein was transferred to the PVDF membrane after electrophoresis and washed with TBST 3 times for 10 min, followed by blocking with 5% bovine serum albumin for 1 h. The membrane was incubated overnight with the corresponding primary antibody against Nrf2 (1 : 2000), HO-1 (1 : 2000), Keap1 (1 : 2000), AKT (1 : 2000), p-AKT (1 : 2000), PI3K (1 : 2000), p-PI3K (1 : 2000), and GAPDH (1 : 20000) at 4°C, followed by incubating with secondary antibody (1 : 20000) at room temperature for 1 h. The chemiluminescence imaging of proteins was performed using ECL reagents (Beijing, Shanghai). The band density was analyzed by using ImageJ software.

### 2.11. Statistical Analysis

Data were analyzed using GraphPad Prism 8.3.0 software. Differences among groups were analyzed by ANOVA. When the *p* value was less than 0.05, it was statistically significant.

## 3. Results

### 3.1. The Effects of Amp-Fed on Histopathological Changes and the Renal Mass Index

**Figure 1:**
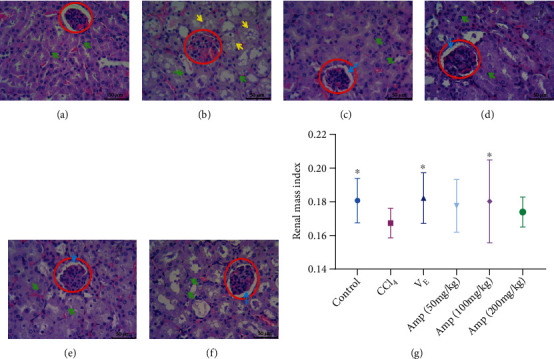
Changes in the kidney pathology (a–f) and the renal mass index (g) in Amp-fed mice with CCl_4_ administration. Control group (a); CCl_4_ group (b); *V*_E_ group (c); 50 mg/kg Amp group (d); 100 mg/kg Amp group (e); 200 mg/kg Amp group (f). H&E staining (400x) with eight mice in each group. Data are expressed as the mean ± SD (*n* = 8 in each group). ^∗^*p* < 0.05, compared to the CCl_4_ group.

In order to observe the pathological change of kidneys in Amp-fed mice with CCl_4_-induced, H&E staining was performed, and the results are shown in Figure 1. Compared with the control group, CCl_4_ caused pathologic alterations of the kidney, including the glomerular swelling (Figure 1(b) area in red circle), vesicular degeneration of epithelial cells (yellow arrow in Figure 1(b)), and nucleus condensation and dissolution (green arrow in Figure 1(b)). The pathological status of the kidney was improved in Amp-fed mice (Figures 1(d)–1(f)). The glomerular swelling (area in red circle) reduced, renal cystic space reappeared (blue arrow), and degeneration and necrosis of renal tubular epithelial cells reduced.

The fresh kidneys of mice were collected and weighed; then the data obtained were used to study the effects of Amp on the kidney weight in CCl_4_ treatment mice (Figure 1(g)). Compared with the control group, the renal mass index of mice was markedly lower in the CCl_4_-treated group. The renal mass index of mice rebounded in *V*_E_ or Amp-fed groups, but there was no significant difference between Amp (50 and 200 mg/kg) groups and the CCl_4_ group.

### 3.2. Effects of Amp-Fed on the Kidney Function

**Figure 2:**
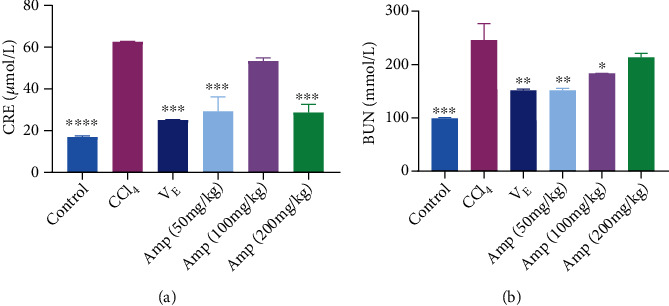
Effects of Amp-fed on CRE (a) and BUN (b) levels in kidney of CCl_4_- induced mice. Data are expressed as the mean ± SD (*n* = 8 in each group). ^∗^*p* < 0.05;  ^∗∗^*p* < 0.01;  ^∗∗∗^*p* < 0.001;  ^∗∗∗∗^*p* < 0.0001, compared to the CCl_4_ group.

As shown in Figure 2, the levels of creatinine (CRE) and urea nitrogen (BUN) in the kidneys of the CCl_4_ group mice were significantly increased compared with those in the control group mice. The levels of CRE (except 100 mg/kg Amp group) and BUN (except 200 mg/kg Amp group) in the kidneys of Amp-fed mice were significantly lower than those of CCl_4_ group mice. The levels of CRE were decreased by over 50% in 50 and 200 mg/kg Amp groups relative to those in the CCl_4_ group. However, it was observed that the level of BUN was inversely proportional to the concentration of Amp.

### 3.3. Effects of Amp-Fed on the Antioxidant Enzyme Activities

**Figure 3:**
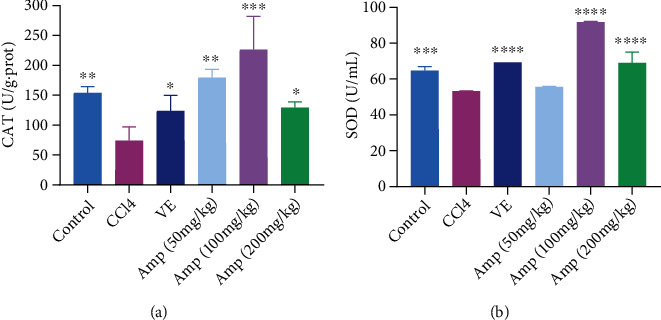
Effects of Amp-fed on the activities of CAT (a) and SOD (b) in the kidney of CCl_4_-treated mice. Data are expressed as the mean ± SD (*n* = 8 in each group). ^∗^*p* < 0.05;  ^∗∗^*p* < 0.01;  ^∗∗∗^*p* < 0.001;  ^∗∗∗∗^*p* < 0.0001, compared to the CCl_4_ group.

The CCl_4_-induced acute kidney injury could lead to the oxidative stress response. In this section, the antioxidant enzyme activities were detected to investigate the preventive effect of Amp-fed on acute kidney injury. Compared with the control group, the levels of catalase (CAT) and superoxide dismutase (SOD) decreased significantly (*p* < 0.01) in the CCl_4_ group. Amp-fed increased the CAT and SOD levels in kidney of CCl_4_-induced mice, but there was no significant difference in SOD levels between the 50 mg/kg Amp group and CCl_4_ group (Figures 3(a) and 3(b)). Furthermore, there was a dependent relationship between the CAT or SOD activity and the concentration of Amp, which increased first and then decreased. The highest levels of CAT and SOD were obtained in the 100 mg/kg Amp group, which were elevated by about three times and 180% relative to those in the CCl_4_ group, respectively.

### 3.4. Effect of Amp-Fed on the Keap1/Nrf2-ARE Signaling Pathway

**Figure 4:**
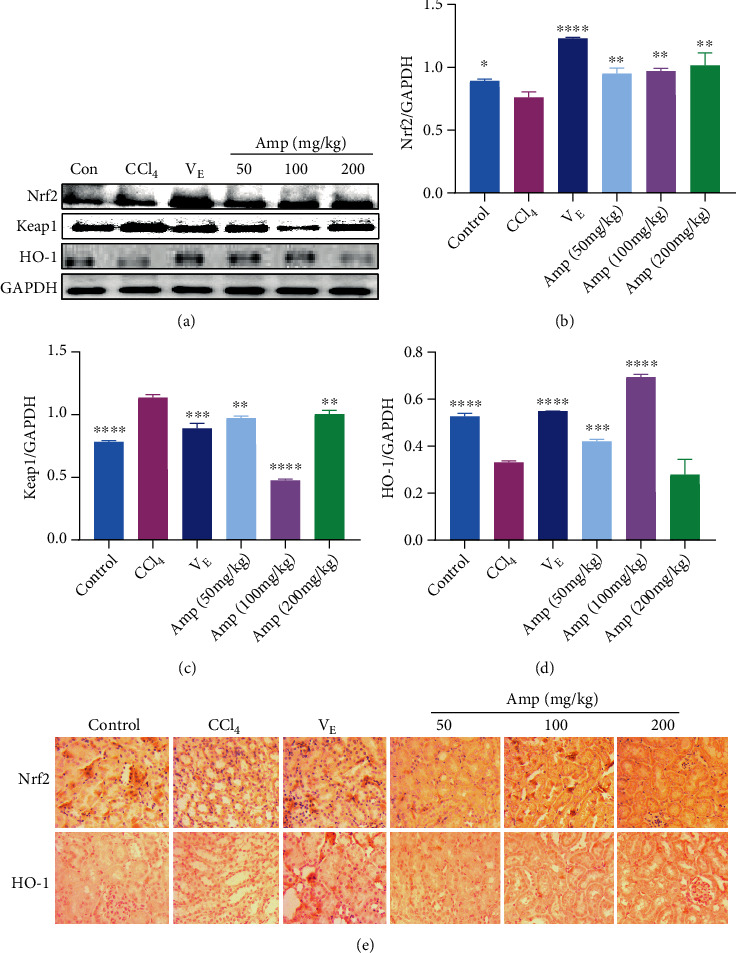
Effect of Amp-fed on the protein levels of the Keap1/Nrf2-ARE signaling pathway. Data are expressed as the mean ± SD (*n* = 8 in each group). ^∗^*p* < 0.05;  ^∗∗^*p* < 0.01;  ^∗∗∗^*p* < 0.001;  ^∗∗∗∗^*p* < 0.0001, compared to the CCl_4_ group.

The Keap1/Nrf2-ARE pathway is one of the important pathways of oxidative stress. In this study, we explored whether Amp plays a protective role through Keap1/Nrf2-ARE pathway by western blot and immunohistochemistry methods. The results of western bolt showed that CCl_4_ treatment significantly increased the level of Keap1 and reduced the level of Nrf2, resulting in downregulation of downstream target protein (antioxidant enzyme HO-1) levels (Figures 4(a)–4(d)). Compared with the control group, Nrf2 levels in Amp-fed groups recovered significantly, but there was no significant difference between the three dose groups of Amp (Figure 4(b)). The Keap1 levels of the three Amp groups decreased significantly relative to those in the CCl_4_ group. However, there was no linear relationship between Keap1 levels and Amp concentrations. The level of Keap1 in the 200 mg/kg Amp group was higher than that in the 100 mg/kg group (Figure 4(c)). Correspondingly, the HO-1 (Figure 4(d)) levels increased significantly in Amp-fed groups. The levels of HO-1 in the 50 mg/kg and 100 mg/kg Amp group were elevated to 158% and 267%, respectively, but it was decreased in the 200 mg/kg Amp group. In the immunohistochemistry (Figure 4(e)), the expressions of Nrf2 and HO-1 were upregulated in the Amp-fed groups, which were consistent with the results of the western bolt.

### 3.5. Effect of Amp-Fed on the Levels of Inflammation-Related Proteins

**Figure 5:**
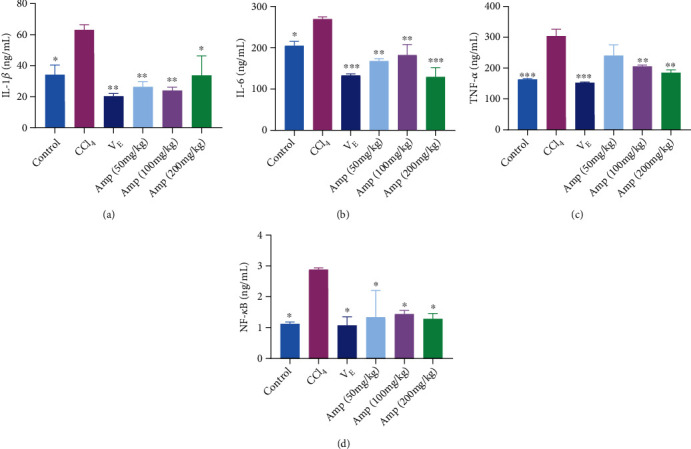
Effect of Amp-fed on the levels of IL-1*β* (a), IL-6 (b), TNF-*α* (c), and NF-*κ*B (d) in CCl_4_-induced mice. Data are expressed as the mean ± SD (*n* = 8 in each group). ^∗^*p* < 0.05;  ^∗∗^*p* < 0.01;  ^∗∗∗^*p* < 0.001;  ^∗∗∗∗^*p* < 0.0001, compared to the CCl_4_ group.

Several major molecules that participate in the inflammation were measured to evaluate whether Amp-fed could prevent CCl_4_-induced kidney damage by inhibiting inflammation. Serum levels of IL-1*β*, IL-6, and TNF-*α* were significantly increased after treatment with 10% CCl_4_ for 16 h, while Amp-fed significantly reduced the number of these inflammatory cytokines (Figures 5(a)–5(c)). These factors are closely related to the activation of NF-*κ*B.  Figure 5(d) indicates that the level of NF-*κ*B was significantly increased in the kidney of mice after CCl_4_ treatment. However, this effect was significantly suppressed in Amp-fed mice. The levels of NF-*κ*B in Amp-fed groups were reduced to only about 50% of that in the CCl_4_ group, but there was no significant difference between the different concentrations of Amp groups.

### 3.6. Effect of Amp-Fed on the PI3K/AKT Pathway

**Figure 6:**
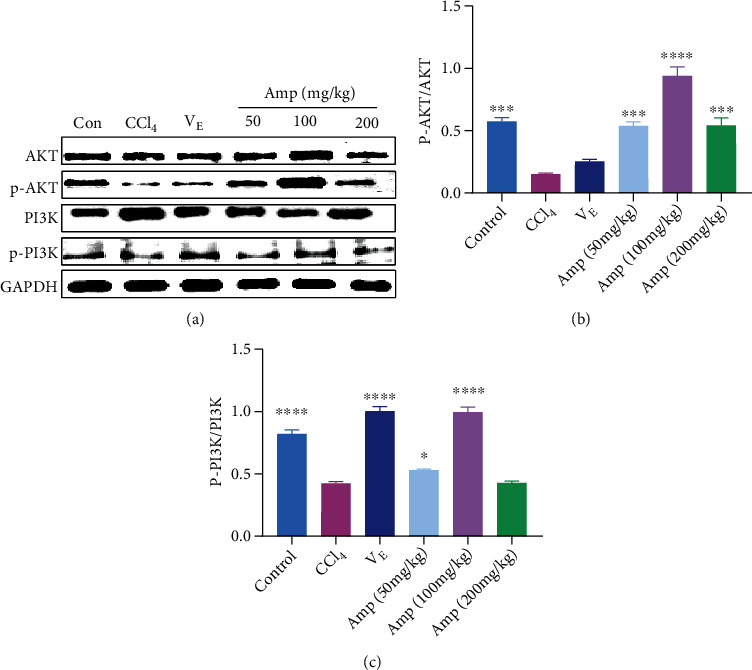
Effect of Amp-fed on the activation of PI3K/AKT pathway in kidney of CCl_4_-induced mice. Data are expressed as the mean ± SD (*n* = 8 in each group). ^∗^*p* < 0.05;  ^∗∗^*p* < 0.01;  ^∗∗∗^*p* < 0.001;  ^∗∗∗∗^*p* < 0.0001, compared to the CCl_4_ group.

The kidney damage caused by CCl_4_ was prevented by Amp-fed via the upregulation of several major molecules that participate in the antioxidant and inflammation pathways. The PI3K/AKT pathway activated by phosphorylation of PI3K and AKT proteins (p-PI3K and p-AKT) is an upstream pathway related to the Keap1/Nrf2 pathway and the NF-*κ*B pathway. In this section, we further investigated whether the PI3K/AKT pathway was involved in the protective effect of Amp on the kidney. As shown in Figure 6, compared with the CCl_4_ group, both PI3K and AKT proteins were activated in the kidney after Amp treatment, especially in the 100 mg/kg group. The values of p-AKT/AKT in the Amp-fed groups were increased to 3.6 times for the 50 mg/kg group, 6.3 times for the 100 mg/kg group, and 3.6 times for the 200 mg/kg group relative to those in the CCl_4_ group. In addition, the values of p-PI3K/PI3K in the 50 and 100 mg/kg of Amp group were also increased to 125% and 234%, respectively.

## 4. Discussion

Acute kidney injury (AKI) is a globally common clinical problem associated with high morbidity and mortality [19]. A variety of events can result in AKI, such as ischemia-reperfusion injury, chemical poisoning, and cardiovascular surgery [20]. The pathophysiology of AKI is multifaceted and involves oxidative stress, inflammation, acute hypoxia, hypoperfusion, and vascular damage [21]. There is growing evidence that oxidative stress plays a key role in the occurrence and development of renal damage in AKI [22, 23]. CCl_4_ is metabolized by the cytochrome P450 enzyme family and subsequently produces highly toxic trichloromethyl and trichloromethyl peroxy free radicals. These free radicals trigger lipid peroxidation and produce more harmful lipid peroxides, leading to AKI.

In this study, the preventive effect and mechanisms of the collagen peptides from *Acaudina molpadioides* (Amp) on AKI induced by CCl_4_ in mice were investigated. Amp-fed significantly improved the decrease in kidney mass index and pathological changes of kidney in mice caused by CCl_4_. These results intuitively indicated that Amp-fed could prevent kidney damage caused by CCl_4_ to a certain extent. The serum urea nitrogen (BUN) and creatinine (CRE) levels are indicators of glomerular filtration rate and renal function [24]. AKI consists of a rapid renal function decline which usually increases BUN and CRE levels. An increase in BUN and CRE levels was observed in the CCl_4_ group compared with those in the control group, suggesting that CCl_4_ caused renal function impairment and renal damage, as shown in previous studies [25]. The levels of BUN and CRE were significantly decreased in Amp groups, which further showed that Amp had a protective effect on kidney damage induced by CCl_4_.

Oxidative damage is one of the typical pathophysiological features of AKI, which contributes to the pathogenesis of AKI [26]. The levels of SOD, CAT, and HO-1 are the key indicators to evaluate the ability of the antioxidant defense system of cells [27]. Collagen peptide pretreatment has previously been shown to suppress intracellular oxidative damage by increasing the levels of antioxidant enzymes [8, 28]. In addition, our previous study has reported that Amp could attenuate oxidative damage induced by H_2_O_2_ in RAW264.7 cells via elevating the activities of SOD and CAT [18]. This prompted us to explore whether Amp could reduce the levels of antioxidant enzymes in the kidney of AKI mice. The results showed that Amp-fed reversed the decrease in SOD, CAT, and HO-1 levels in the kidney of CCl_4_-induced AKI mice (Figures 3 and 4(d)), suggesting that Amp may significantly reduce the oxidative stress induced by CCl_4_ in mouse kidneys. However, it was observed that their levels were lower in the 200 mg/kg Amp group than those in the 100 mg/kg group, which may be due to the renal toxicity of high concentrations of Amp. These results were consistent with the results of Amp on the renal mass index (Figure 1(g)).

The Nrf2 signaling pathway is an important oxidative stress defense response system that regulates the expression of downstream antioxidant enzyme genes [23, 29]. Under normal physiological conditions, the complex of Keap1-Nrf2 is in the cytoplasm. Nrf2 is activated once it disengages from Keap1 and enters the nucleus. In the present study, Amp-fed significantly upregulated the expression of Nrf2 and downregulated the levels of Keap1 relative to the CCl_4_ group, indicating that more Nrf2 were detached from Keap1 and thus activated. These results suggested that Amp may be involved in the inhibition of CCl_4_-induced oxidative stress in the kidneys by activating the Nrf2 signaling pathway. However, Keap1 level was elevated in the 200 mg/kg Amp group compared to that in the 100 mg/kg group. Higher level of Keap1 may lead to inhibition of Nrf2 activation, thereby downregulating the levels of downstream antioxidant enzymes. This was evidenced by the lower levels of SOD, CAT, and HO-1 in the 200 mg/kg Amp group (Figures 3 and 4(d)). Our results are consistent with a previous study wherein the AKI was attenuated by elevating antioxidative status via Nrf2 pathway activation [8, 30].

Inflammation is a fundamental response in the pathogenesis of AKI. The oxidative stress stimulates the inflammatory response mediated by the NF-*κ*B signaling pathway, which regulates the levels of inflammatory mediators such as IL-1*β*, IL-6, and TNF-*α* [31, 32]. In the current study, Amp-fed prevented CCl_4_-induced renal IL-1*β*, IL-6, TNF-*α*, and NF-*κ*B elevation. These data demonstrated that Amp revealed the anti-inflammatory effect by reducing the levels of IL-1*β*, IL-6, and TNF-*α* in the CCl_4_-induced AKI mice, and NF-*κ*B might be the beneficial pathway for this anti-inflammatory process. Over the years, various compounds have been shown as a mediator of inflammation in AKI. Dexmedetomidine ameliorated lipopolysaccharide-induced AKI in rats by inhibiting inflammation. Hydrogen sulfide attenuated LPS-induced AKI by inhibiting inflammation. In addition, collagen peptides from the fish scales have been reported to suppress the levels of IL-1*β* and TNF-*α* by inhibiting NF-*κ*B activation in HaCaT cells, thereby inhibiting inflammation [33].

PI3K/AKT is an important pathway involved in oxidative damage and inflammation. PI3K and AKT proteins activate or inhibit the activity of a series of downstream substrates through phosphorylation, thereby participating in cellular processes such as oxidative stress and inflammation. It has been reported that PI3K/AKT is one of the upstream pathways regulating the Nrf2 and NF-*κ*B pathway, and many compounds could activate the Nrf2 or NF-*κ*B pathway through the PI3K/AKT to protect cells or tissues from damage [8, 9, 34]. In the present study, the phosphorylation of PI3K and AKT was upregulated significantly in the Amp-fed groups, relative to the CCl_4_ group, suggesting that Amp could activate the PI3K/AKT pathway. It was reported that collagen peptides from skate (*Raja kenojei*) skin could stimulate the phosphorylation of PI3K and AKT by upregulating the phosphorylation of insulin receptor substrate (IRS) protein [8]. The mechanism of activation of PI3K/AKT pathway by Amp might be similar to that of collagen peptides from skate (*Raja kenojei*) skin, but further verification is required.

**Figure 7:**
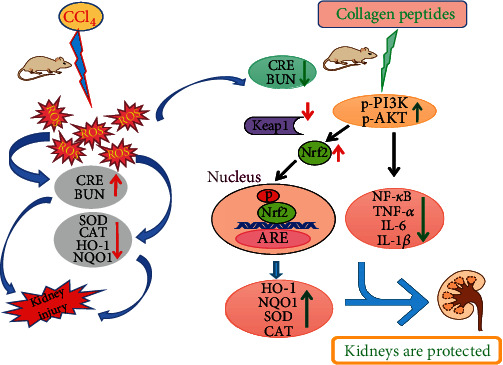
Molecular mechanism of Amp's protective effect on CCl_4_-induced acute kidney injury through PI3K/AKT-mediated Nrf2 and NF-*κ*B signaling pathways.

In summary, Amp could activate the Nrf2 pathway, thereby reversing the decline in antioxidant enzymes levels caused by CCl_4_. In addition, Amp could also exert an anti-inflammatory effect and reduce the acute injury of CCl_4_ to the kidney by activating the NF-*κ*B signaling pathway. The activation of Nrf2 and NF-*κ*B signaling pathways may be related to the activation of PI3K/AKT signaling pathway by Amp (Figure 7). According to literature reports, propolis can protect CCl_4_-induced kidney injury by modulation of oxidative parameters [35]. Ellagic acid prevents kidney injury and oxidative damage via regulation of Nrf2/NF*κ*B signaling in CCl_4_-induced rats [36]. Collagen peptides from the skin of monkfish (*Lophius litulon*) ameliorate kidney damage in high-fat diet fed mice through antioxidant and anti-inflammatory mediated by Nrf2 and NLRP3 signaling pathway [37]. However, whether they can activate the PI3K/AKT pathway has not been explored. In the present study, collagen peptides from sea cucumber *Acaudina molpadioides* prevent AKI through antioxidant and anti-inflammatory mediated by PI3K/AKT/Nrf2 and PI3K/AKT/NF-*κ*B pathway, respectively. However, the specific targets and regulatory mechanisms of Amp have not yet been clarified. In addition, the structure-activity relation of the peptide chains in Amp is unclear. In the future, we will focus on solve these problems by using pathway inhibitors and identifying the activity of each peptide component in Amp.

## 5. Conclusion

In this study, we observed that Amp-fed effectively increased renal mass index, improved pathological morphology of kidneys, and decreased BUN and CRE levels in AKI mice. In addition, Amp-fed induced a decrease in markers of oxidative stress and inflammatory response. These results indicated that Amp protected against CCl_4_-induced AKI via attenuation of oxidative stress and inflammatory response. The protective effect of Amp may be attributable to activate Nrf2-mediated antioxidant and NF-*κ*B-mediated anti-inflammatory pathways, both of which were regulated through the PI3K/AKT pathway. Taken together, these findings suggested that Amp is expected to prevent AKI caused by CCl_4_.

## Data Availability

The date used to support the findings of this study are included within the article.
